# Reversal Activity and Toxicity of Heparin-Binding Copolymer after Subcutaneous Administration of Enoxaparin in Mice

**DOI:** 10.3390/ijms222011149

**Published:** 2021-10-15

**Authors:** Justyna Swieton, Joanna Miklosz, Shin-Ichi Yusa, Krzysztof Szczubialka, Dariusz Pawlak, Andrzej Mogielnicki, Bartlomiej Kalaska

**Affiliations:** 1 Department of Pharmacodynamics, Medical University of Bialystok, Mickiewicza 2c, 15-089 Bialystok, Poland; justyna.swieton@gmail.com (J.S.); joanmiklosz@gmail.com (J.M.); dariusz.pawlak@umb.edu.pl (D.P.); andrzej.mogielnicki@umb.edu.pl (A.M.); 2 Department of Applied Chemistry, Graduate School of Engineering, University of Hyogo, 2167 Shosha, Himeji 671-2280, Japan; yusa@eng.u-hyogo.ac.jp; 3 Faculty of Chemistry, Jagiellonian University, Gronostajowa 2, 30-387 Krakow, Poland; k.szczubialka@uj.edu.pl

**Keywords:** anticoagulation, antidote, block copolymer, enoxaparin, heparins, macromolecules, mice, protamine, rodents, toxicity

## Abstract

Uncontrolled bleeding after enoxaparin (ENX) is rare but may be life-threatening. The only registered antidote for ENX, protamine sulfate (PS), has 60% efficacy and can cause severe adverse side effects. We developed a diblock copolymer, heparin-binding copolymer (HBC), that reverses intravenously administered heparins. Here, we focused on the HBC inhibitory activity against subcutaneously administered ENX in healthy mice. BALB/c mice were subcutaneously injected with ENX at the dose of 5 mg/kg. After 110 min, vehicle, HBC (6.25 and 12.5 mg/kg), or PS (5 and 10 mg/kg) were administered into the tail vein. The blood was collected after 3, 10, 60, 120, 360, and 600 min after vehicle, HBC, or PS administration. The activities of antifactors Xa and IIa and biochemical parameters were measured. The main organs were collected for histological analysis. HBC at the lower dose reversed the effect of ENX on antifactor Xa activity for 10 min after antidote administration, whereas at the higher dose, HBC reversed the effect on antifactor Xa activity throughout the course of the experiment. Both doses of HBC completely reversed the effect of ENX on antifactor IIa activity. PS did not reverse antifactor Xa activity and partially reversed antifactor IIa activity. HBC modulated biochemical parameters. Histopathological analysis showed changes in the liver, lungs, and spleen of mice treated with HBC and in the lungs and heart of mice treated with PS. HBC administered in an appropriate dose might be an efficient substitute for PS to reverse significantly increased anticoagulant activity that may be connected with major bleeding in patients receiving ENX subcutaneously.

## 1. Introduction

Enoxaparin (ENX) belongs to the low-molecular-weight heparins (LMWHs). It inhibits factors Xa and IIa activities with a 4:1 ratio [1] and is used in angina, acute coronary, and venous thromboembolism treatment and prophylaxis [2]. LMWHs offer the advantage of once-daily subcutaneous administration, which improves patient compliance. Routine plasma monitoring is not needed due to their predictable anticoagulant effect [3]. Although their clearance is easy to predict and mostly kidney-dependent, increased bleeding may occur in patients without monitoring of antifactor Xa activity with signs of liver and kidney dysfunction, advanced age, history of bleeding, or undergoing surgery [4].

Protamine sulfate (PS), the only specific antidote for reversal of heparin activities, is partially effective against LMWHs and may cause life-threatening hypersensitivity reactions [5]. Many studies were undertaken to obtain PS substitutes. Andexanet alfa [6], ciraparantag (PER977) [7], and universal heparin reversal agent (UHRA) [8] are currently in the preclinical or clinical trials. The development of many alternative antidotes such as hexadimethrine bromide (polybrene) [9], low molecular weight protamine [10], chemically modified inactive antithrombin [11], or delparantag (PMX-60056) [12] was suspended because of unacceptable side effects. We have developed a new synthetic compound, heparin-binding copolymer (HBC), that efficaciously binds and completely neutralizes unfractionated heparin (UFH) and LMWHs both in vitro and in vivo [13,14]. Furthermore, besides mild and occasional clinical signs of toxicity and gross necropsy findings, its single therapeutic dose of 20 mg/kg was well tolerated by rats [14].

Here, we have evaluated the timeline of HBC inhibitory activity against ENX in healthy mice. Simultaneously, we have determined organ toxicity and biochemical parameters to develop an efficient and safe protocol for reversing the anticoagulant activity of subcutaneously administered LMWHs.

## 2. Results

### 2.1. Reversing Antifactors Xa and IIa Activities

**Figure 1:**
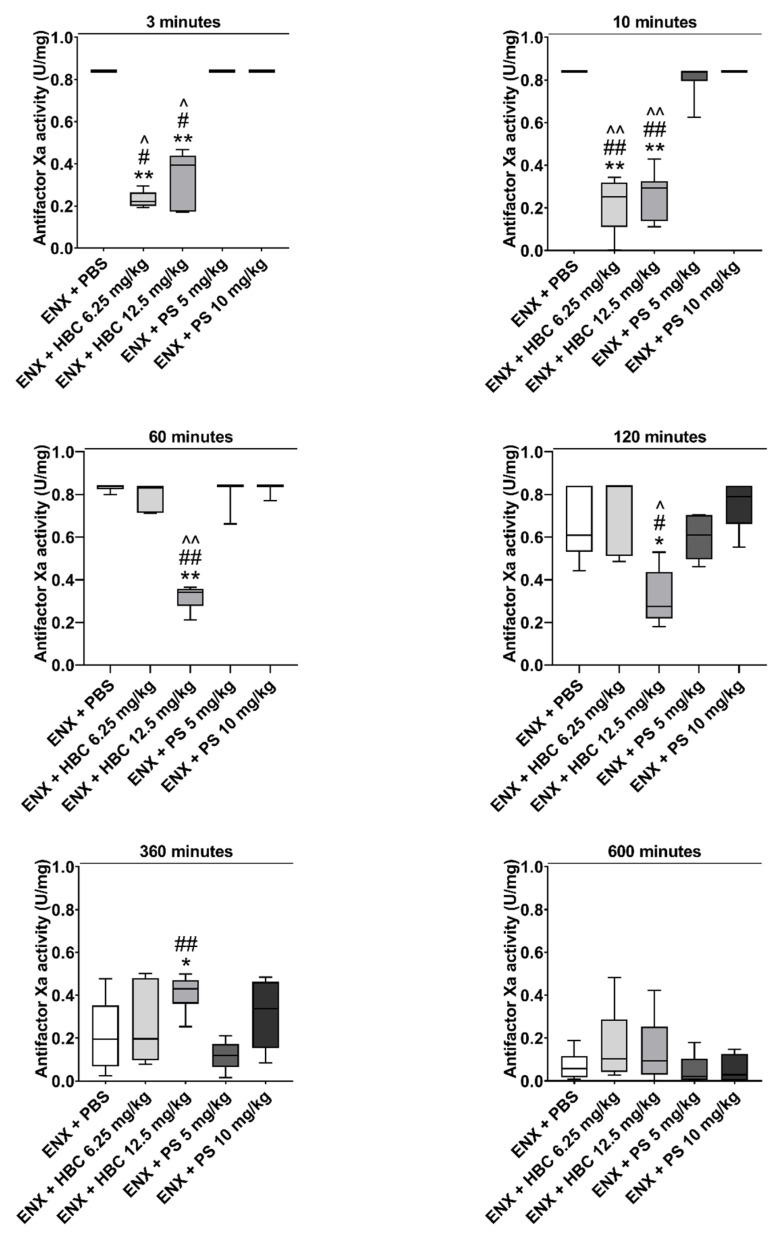
The time-course of the neutralization of enoxaparin (ENX) by HBC (6.25 and 12.5 mg/kg) or protamine sulfate (PS) (5 and 10 mg/kg) in mice, measured by antifactor Xa activity 3, 10, 60, 120, 360, and 600 min after antidote administration. * *p* < 0.05, ** *p* < 0.01 vs. ENX + PBS (phosphate buffered saline), # *p* < 0.05, ## *p* < 0.01 vs. PS 5 mg/kg, ^ *p* < 0.05, ^^ *p* < 0.01 vs. PS 10 mg/kg, Mann–Whitney test. Results are shown as median with lower and upper limits, *n* = 6.

**Figure 2:**
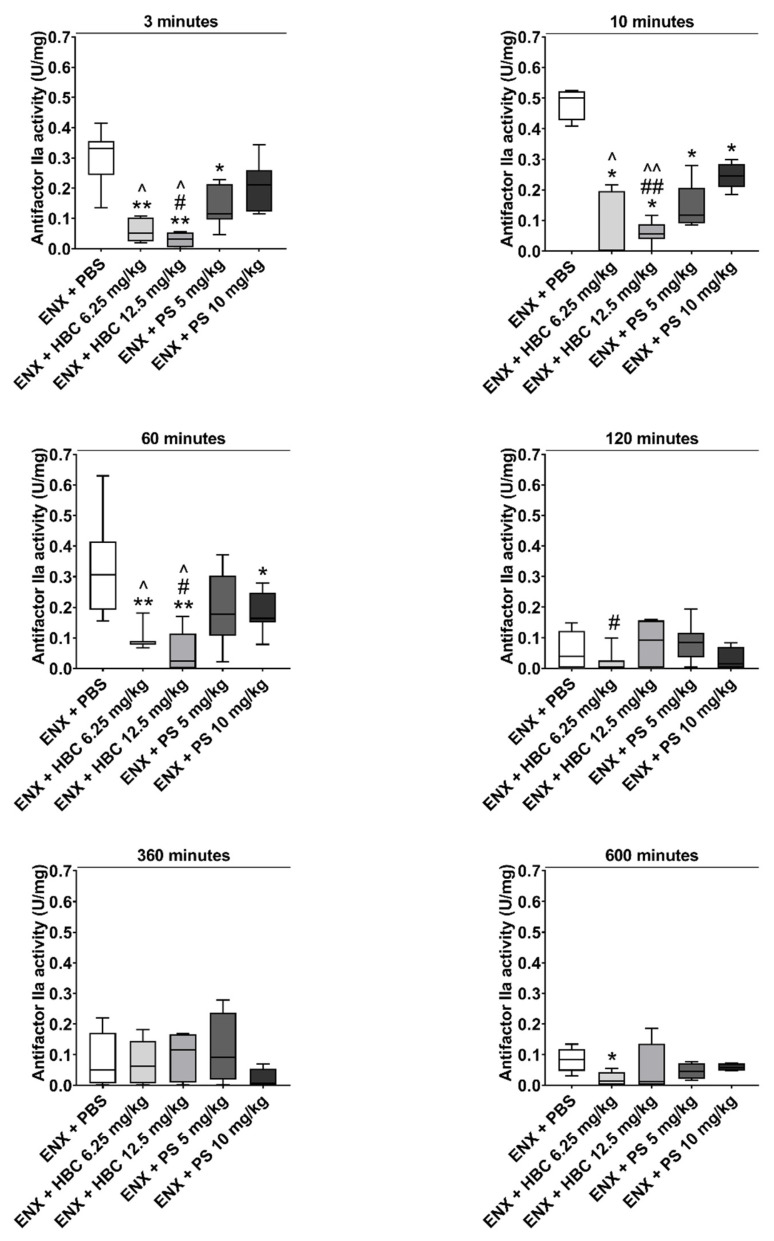
The time-course of neutralization of enoxaparin (ENX) by HBC (6.25 and 12.5 mg/kg) or protamine sulfate (PS) (5 and 10 mg/kg) in mice measured by antifactor IIa activity 3, 10, 60, 120, 360, and 600 min after antidote administration. * *p* < 0.05, ** *p* < 0.01 vs. ENX + PBS (phosphate buffered saline), # *p* < 0.05, ## *p* < 0.01, vs. PS 5 mg/kg, ^ *p* < 0.05, ^^ *p* < 0.01 vs. PS 10 mg/kg, Mann–Whitney test. Results are shown as median with lower and upper limits, *n* = 6.

HBC at the lower dose reversed antifactor Xa activity 3 and 10 min after administration. HBC administered at the higher dose reversed antifactor Xa activity throughout the course of the experiment. During the first 2 h after administration, HBC at the higher dose showed almost complete reversal of antifactor Xa activity. In contrast, PS at both doses did not reverse antifactor Xa activity increased by ENX at any of the studied time points (Figure 1). HBC at both doses completely reversed the effect of ENX on antifactor IIa activity at each time point. PS at both doses partially neutralized antifactor IIa activity increased by ENX, although PS at the lower dose was effective only 3 and 10 min after administration (Figure 2).

### 2.2. Organs Morphology

Moderate vacuolation of tunica media myocytes in kidney arteries was found in both HBC groups and PS at the lower dose, whereas vacuolation of convoluted tubule epithelial cells was found in groups treated with HBC and PS at the higher doses.

The histopathological examination of lungs showed moderate to severe changes in perivascular mononuclear cell infiltrate, alveolar edema, and congested bronchioles in all studied groups, but the alterations were slightly more abundant in groups treated with HBC at the lower dose and PS at the higher dose.

Concerning the heart tissue, PS at the higher dose significantly increased the frequency and severity of myofiber waviness and multifocal necrosis.

Liver injuries perceived in mice treated with HBC at the higher dose ranged from hepatocellular hypertrophy, necrosis of hepatocytes, and neutrophilic infiltration to congestion. Each histological alteration in the liver applied to fifty percent of mice treated with HBC at the higher dose.

**Figure 3:**
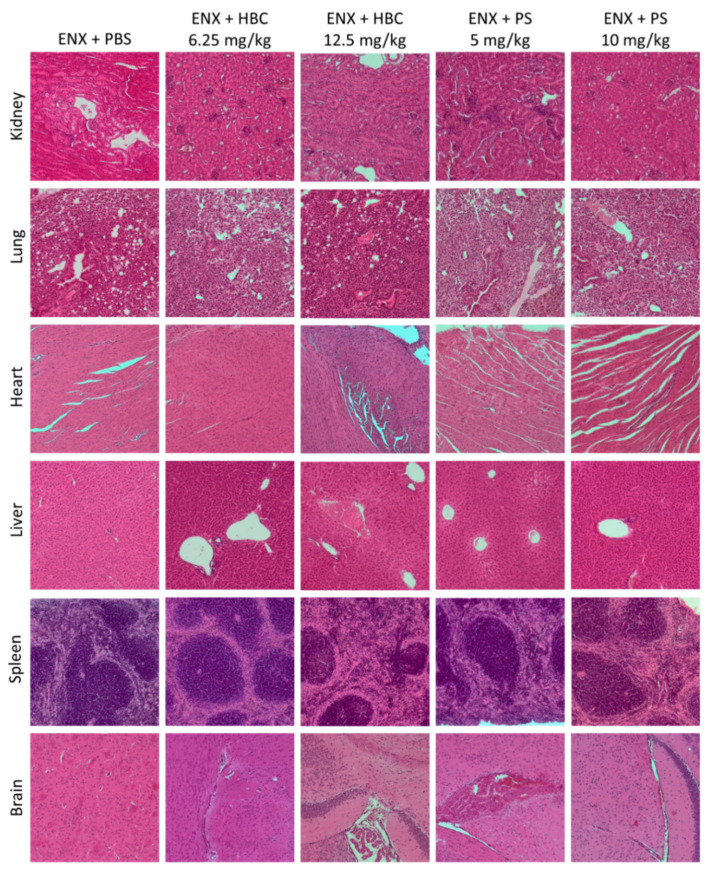
Representative micrographs of tissue sections of kidney, lung, heart, liver, spleen, and brain sections of mice treated subcutaneously with enoxaparin (ENX) (5 mg/kg) and an intravenous bolus of vehicle, HBC (6.25 and 12.5 mg/kg) or protamine sulfate (PS) (5 and 10 mg/kg). Organs were harvested 10 h after antidote administration. Original magnification, 100×; hematoxylin and eosin staining. PBS, phosphate buffered saline.

**Figure 4:**
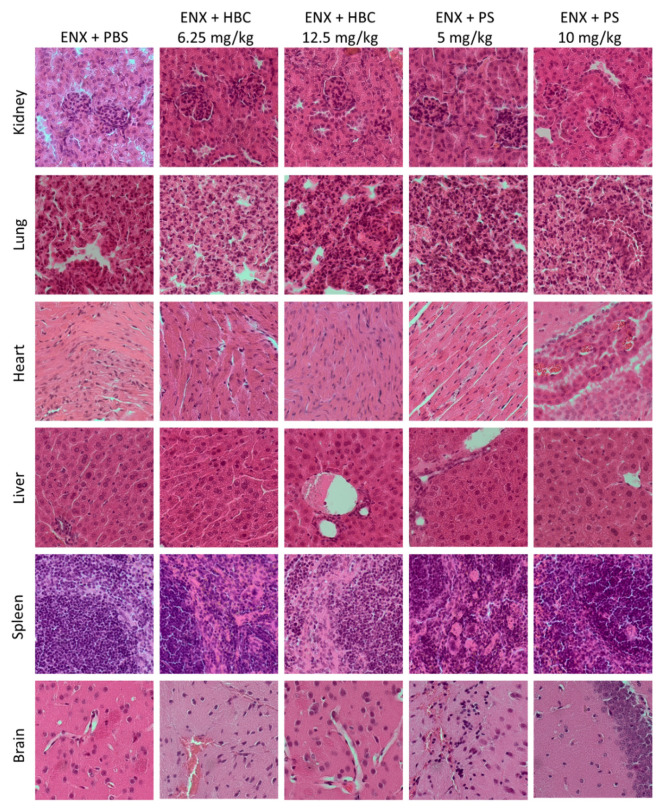
Representative micrographs of tissue sections of kidney, lung, heart, liver, spleen, and brain sections of mice treated subcutaneously with enoxaparin (ENX) (5 mg/kg) and an intravenous bolus of vehicle, HBC (6.25 and 12.5 mg/kg) or protamine sulfate (PS) (5 and 10 mg/kg). Organs were harvested 10 h after antidote administration. Original magnification, 400×; hematoxylin and eosin staining. PBS, phosphate buffered saline.

Moderate to severe degenerative changes in the spleen such as decreased cellularity in the red and white pulp, increased apoptosis of lymphocytes in mantel zone, increased cellularity of fibroblastic reticular cells (FRCs) and macrophages in red pulp/stroma, connective tissue hyperplasia/deposition of collagen fibers in red pulp/stroma, and an increase in megakaryocytes in the red pulp were more pronounced in the group treated with HBC at the higher dose (Table 1, Figure 3 and Figure 4).

### 2.3. Biochemical Parameters

HBC at the lower dose increased alanine transaminase (ALT) activity and albumin concentration. Mice exposed to HBC at the higher dose revealed significantly increased serum liver transaminases and lactate dehydrogenase (LDH) activities, cholesterol, albumin, and globulin concentrations as well as elevated creatine kinase (CK) levels, with a decline in potassium in comparison with the vehicle group. No alteration in parameters was found in mice treated with PS at the lower dose. A significant increase in ALT activity was shown in mice treated with PS at the higher dose compared with the vehicle group (Table 2).

## 3. Discussion

Our previous studies demonstrated that HBC reverses the activity of UFH and LMWHs. The complete HBC reversal effect was observed after intravascular administration of heparins in rats [13,14], pointing to its superiority over PS. The present study aimed to evaluate the activity of HBC against ENX administered subcutaneously at several time points to determine an optimal dosage regimen for reversing of the anticoagulant activity of LMWHs. We found that HBC at the higher dose completely neutralized ENX, maintaining low values of antifactor Xa and IIa activities throughout the whole experiment. Although HBC was generally well-tolerated, increasing a dose equal to the mass ratio of HBC to ENX above 2:1 revealed potential targets of its toxicity.

Anticoagulant therapy pursues optimal therapeutic effect while minimizing the adverse reactions of the treatment at the same time. Despite high effectiveness, bleeding complications after ENX treatment occur [15,16,17,18]. To reflect the clinical scenario of bleeding, we used ENX in the supratherapeutic dose of 5 mg/kg to enhance the anticoagulant effect in mice. For the treatment of venous thromboembolism and acute coronary syndrome in humans, the recommended subcutaneous dose is 30 mg/day or 1 mg/kg/day [19]. In the case of major bleeding, the antidote for anticoagulants should be administered intravenously for immediate reaction. The precise dosing of the antidote is clinically significant, as it can influence anticoagulant properties, and the excess of an antidote may contribute to bleeding [20]. PS administered alone can exhibit anticoagulant activity, inhibit thrombin and factor VII activity, impair platelet function, and negatively impact lungs and heart [21,22]. Those were not reported when PS was administered with UFH, which might bind PS and thus counter its effect [23].

In our previous study [13], both antifactor Xa activity and bleeding time were measured after intravenous ENX administration at the dose of 10 mg/kg and infusion of HBC or PS at the effective ratios. We have demonstrated that HBC stops bleeding induced by ENX in rats more effectively and significantly than PS. Based on the previous results, we used 6.25 and 12.5 mg/kg of HBC, corresponding to a HBC/ENX mass ratio of 1.25:1 and 2.5:1, respectively, and 5 and 10 mg/kg of PS, corresponding to a PS/ENX mass ratio of 1:1 and 2:1, respectively [13,14]. In the present study, HBC at the lower dose completely reversed the antifactor Xa activity for 10 min and maintained low antifactor IIa activity throughout the whole experiment, whereas PS at both doses showed significantly weaker activities against those two factors. The time-course of antifactor Xa activity induced by PS reflects the changes observed after ENX administration, indicating a poor reversal of ENX by PS, in accordance with the literature [24]. HBC administered at the lower dose could be a practical tool when the effect of ENX is descending, or when immediate and complete neutralization would have harmful consequences for health, in patients having a high risk of thrombosis, or experiencing multiple comorbidities associated with a higher risk of stroke or heart attack [25,26,27,28]. HBC administered at the higher dose had a rapid and long-term effect. The antifactor Xa activity was almost completely reduced and maintained until ENX activity was no longer visible, except slightly increased antifactor Xa activity in 360 min after HBC administration. This may indicate the anticoagulant properties of cationically modified polymers at high doses, as we showed in a previous study [29]. Based on the literature, polycations can possess anticoagulant activity when used in high concentrations/doses or in excess of polyanions. Their anticoagulant activity can be attributed to an interaction with platelet function, activation of coagulation factors, and potentiation of fibrinolysis [20,30]. However, understanding of the specific mechanism of anticoagulant activity after exposure to polycations remains incomplete. Nevertheless, such high doses of HBC should not be expected to be applied under clinical conditions. The antifactor IIa activity was also completely reduced throughout the whole experiment. In the case of emergency bleeding after heparins, HBC would be a valuable reversal agent, as one bolus reversed the antifactor Xa activity similar to the polycationic antidote universal heparin reversal agent (UHRA) [8]. Other antidotes already tested in clinical trials with a long-duration response to LMWHs are ciraparantag and andexanet alfa. One bolus of ciraparantag almost completely normalized ENX-prolonged clotting time for 8 h after antidote administration. Even longer activity in patients was shown by andexanet alfa, which maintained antifactor Xa activity at the control value for 12 h after ENX administration. However, in the clinical trial NCT02329327, a 2-h infusion was necessary after bolus administration to sustain high inhibitory activity.

Polycations may differ in activity depending on whether they are administered alone or with an excess of polyanions. HBC toxicity seems to depend not only on its dose but also on the presence of heparins and animal species tested. In our previous study, rats were monitored for up to 4 days after HBC administration alone for clinical signs and histopathology. HBC did not induce any severe clinical and gross necropsy alternations at the dose of up to 20 mg/kg. HBC at the dose of 40 mg/kg influenced rat kidney function with a residual impact on the liver. HBC also induced apathy and decreased locomotive activity in animals and increased thirst, porphyrin secretion, and mild allergic reactions in fewer than 50% of the animals. However, all those changes were transient and resolved within 24 h [14].

In the present study, ENX injection was followed by HBC, but single moderate changes in kidneys occurred, which may suggest excessive binding of cationic HBC with the anionic surface of renal epithelial cells [31,32]. Here, we observed a slight increase in albumin, indicating that the excess of HBC could lead to the decrease of blood volume. Indeed, we have previously observed the osmotic effect of HBC when administered as a fast injection, whereas slow infusion of HBC did not alter the blood pressure [13]. Kidney analysis did not reveal any severe damage after administration of HBC and, therefore, the alteration of blood volume may be transient and not connected with kidney dysfunction. Administration of HBC resulted in mild apathy and, despite unlimited access to water, a decreased amount of water intake. Under clinical conditions, this changed behavior would be omitted due to, e.g., the accessibility of intravenous drip infusion. HBC influenced liver function in a dose-dependent manner. Severe changes in the liver along with elevated levels of hepatic enzymes (ALT and AST) can reflect the hepatotoxicity of the HBC–ENX complexes, although HBC at a lower dose elevated ALT levels mildly, indicating transient failure [33]. Additionally, criteria based on the Drug-Induced Liver Injury Network were used to assess serum ALT level of 1.25–2.5 times normal as Grade 1, indicating mild hepatotoxicity. Polycation–polyanion complexes can cause adverse effects such as inflammation [34,35]. On the other hand, cationic compounds can impair the liver function [36,37,38], interact with anionic cell lipid membranes [39,40], impact clot morphology [41,42], lyse erythrocytes [43], generate cellular [44] and hematological [45,46] toxicity, and increase LDH levels associated with inflammatory disorders [47]. Therefore, it is not clear if hepatotoxicity is associated directly with the HBC–ENX complexes or the excess of polycationic HBC. An increased number of neutrophils in the liver was observed along with a decrease of lymphocytes in the spleen. Since neutrophils can modulate the lymphocytes to initiate immune responses [48], the alteration in the number of lymphocytes and neutrophils may indicate inflammation in response to HBC.

HBC and PS at both doses induced single cases of moderate to severe changes in lungs. We and others previously reported that polycations may induce lung congestion [49] and mononuclear cell infiltration [50]. PS impaired heart tissue, and it was previously reported to alter the cardiac function of zebrafish and the heart rate of rats [51] by directly affecting myocyte contractile functions [52,53]. We have also found some histological changes in the brain, the spleen, and other organs that occurred in all animals, including in the vehicle group, indicating that they were related to the experimental procedures. Despite observed abnormalities, other studies on using polycations as antidotes indicated their satisfactory biocompatibility. In the safety study of UHRA, the maximum dose of 50 mg/kg (over 16 times higher than the effective dose) was not lethal, and no damage to organs was found 29 days after drug administration. Additionally, ciraparantag caused no deaths or serious adverse effects in the study with patients [7].

We presented an antidote for the neutralization of anticoagulants administered intravenously and subcutaneously [13,14] that is more efficient than PS. The activity of HBC at the lower dose seems to be rapid, but short in duration. The decrease in inhibition of antifactor Xa activity by HBC could result from the excess of ENX or differences in the pharmacokinetics of ENX and HBC. The reversal activity of HBC at the higher dose after a single injection was maintained at all time points. The safety experiments indicated the lowering of HBC dose may be needed depending on the time from LMWHs administration and its dose. However, the dose of ENX subcutaneously administered to mice in our study was rather high and corresponded to overdose in patients. Since the ratio of HBC/ENX is around 2.5:1 [14], the dose of HBC needed to reverse the effect of ENX would be probably several times lower and display fewer to no adverse effects. Previously, HBC neutralized the activity of all heparin-based anticoagulants administered intravenously [14], and in the present work, it also neutralized LMWHs administered subcutaneously. The effect of HBC lasted until the anticoagulant activity of ENX was visible in antifactor Xa and IIa assays. The toxicity study assessed dose-dependent adverse effects; hereby, dosage adjustment should be based on the compromise between reaching the desired activity and maintaining maximum safety.

## 4. Materials and Methods

### 4.1. Materials

ENX was purchased from Sanofi-Aventis, Gentilly, France. PS, Grade X, was purchased from Sigma-Aldrich, Darmstadt, Germany. Phosphate buffered saline (PBS) and trisodium citrate (≥99%) were purchased from Sigma-Aldrich, Darmstadt, Germany and isoflurane was purchased from Baxter, Unterschleißheim, Germany. Antifactor Xa and IIa assay kits were purchased from BioMedica Diagnostic, Windsor, NS, Canada. Lidocaine spray 10% was purchased from Egis-Pharmaceutical, Budapest, Hungary, and 10% buffered formalin was purchased from Chempur, Piekary Slaskie, Poland. HBC was synthesized as previously described [13].

### 4.2. Animals

Male and female BALB/c mice were obtained from the Centre of Experimental Medicine in the Medical University of Bialystok. Animals were housed with a 12 h light/dark cycle in temperature (22 ± 2 °C) and humidity (55 ± 10%) controlled room, grouped into cages as appropriate, and allowed to have ad libitum access to sterilized tap water and standard chow. All the procedures involving animals were approved by the Local Ethical Committee on Animal Testing (Permit Number 07/2021) and conducted in accordance to the Directive 2010/63/EU of the European Parliament and of the Council on the protection of animals, Animal Research: Reporting of In Vivo Experiments guidelines, and national laws. All animals were euthanized by exsanguination at the end of the experiments.

### 4.3. Reversing Antifactors Xa and IIa Activities

Ninety male and ninety female BALB/c mice were randomly assigned to thirty groups. Female body weight was 19.8 ± 2.4 g, whereas male body weight was 25.4 ± 3.4 g. ENX (5 mg/kg, 4 mL/kg) was administered subcutaneously in bolus. After 110 min, vehicle (PBS), HBC (6.25 or 12.5 mg/kg, 4 mL/kg) or PS (5 or 10 mg/kg, 4 mL/kg) were administered into the tail vein. Lidocaine spray was used for reducing pain. All animals were anaesthetized with a mixture of isoflurane and oxygen (3% *v*/*v*), and placed in the anesthetic induction chamber, equipped with an individual mask providing a mixture of isoflurane and oxygen (1.5–2% *v*/*v*). The blood from the heart was collected after 3, 10, 60, 120, 360, and 600 min after PBS, HBC, or PS administration (6 mice per time point). Samples were collected in tubes containing trisodium citrate in a volume ratio of 9:1, centrifuged at 3500× *g* at 4 °C for 20 min, and plasma was deep-frozen (−80 °C) until further assays could be performed. The neutralization of ENX was analyzed by measuring antifactor Xa and antifactor IIa activity in a 96-well plate reader (Synergy HTX, BioTek, Winooski, VT, USA), according to the kit manufacturer instructions (BioMedica Diagnostics, Windsor, NS, Canada), modified as described previously [14].

### 4.4. Analysis of Biochemical Parameters and Organs Morphology

**Figure 5:**
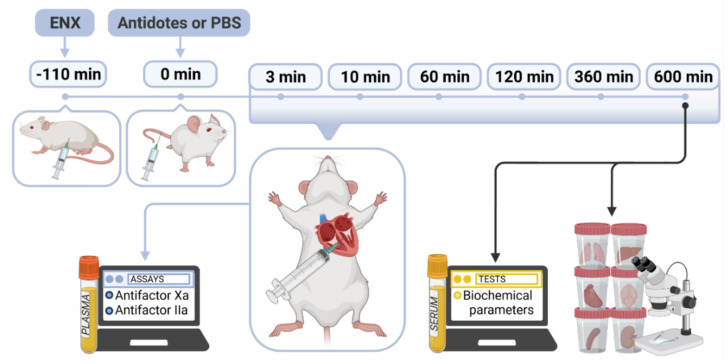
Schematic representation of the study protocol. The diagram shows the time-course of events from enoxaparin (ENX) subcutaneous administration and injection with vehicle (PBS) or antidotes: HBC (6.25 and 12.5 mg/kg) or protamine sulfate (5 and 10 mg/kg) into the mice tail vein.

Biochemical parameters and organs morphology were additionally examined at the last time point (600 min after intravenous boluses of antidotes) of the experiment. Blood samples were put into tubes without anticoagulants and allowed to coagulate, then centrifuged at 10,000× *g* at 20 °C for 5 min. The sera were collected and deep-frozen (−80 °C) for subsequent measurement of ALT, aspartate transaminase (AST), urea, creatinine, albumin, globulin, sodium, potassium, total bilirubin, cholesterol, LDH, triglyceride, and CK. After blood sample collection, mice were euthanized. Four mice from each group were randomly selected for tissue samples collection. Kidney, lung, heart, liver, spleen, and brain were collected and fixed in 10% buffered formalin for routine histological analysis using hematoxylin and eosin staining. The study protocol was presented in Figure 5.

### 4.5. Statistical Analysis

For each test, the experimental unit was an individual animal. The Shapiro–Wilk test was used for data distribution analysis. The non-Gaussian data were presented as median with lower and upper limits using the nonparametric Mann–Whitney test. The data were analyzed with GraphPad Prism 8 (GraphPad Software, La Jolla, CA, USA). The results were graphically presented using BioRender (Toronto, ON, Canada) or GraphPad Prism 8. *p*-values less than 0.05 were considered significant.

## Figures and Tables

**Table 1:** Summary of histological changes in the kidney, lung, heart, liver, and spleen of mice treated subcutaneously with enoxaparin (ENX) (5 mg/kg) and an intravenous bolus of vehicle, HBC (6.25 and 12.5 mg/kg), or protamine sulfate (PS) (5 and 10 mg/kg). The organs were harvested 10 h after antidote administration.

	ENX + PBS	ENX + HBC 6.25 mg/kg	ENX + HBC 12.5 mg/kg	ENX + PS 5 mg/kg	ENX + PS 10 mg/kg
Kidney					
Vacuolation of tunica media myocytes in kidney arteries	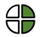	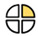	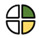	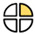	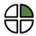
Cortical vacuolation	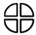	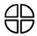	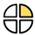	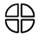	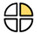
Lung					
Perivascular mononuclear cell infiltrate	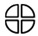	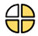	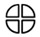	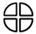	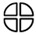
Alveolar edema	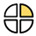	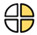	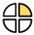	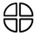	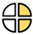
Congested bronchioles	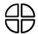	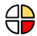	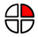	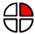	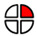
Heart					
Myofiber waviness	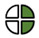	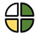	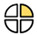	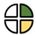	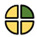
Cardiomyocyte necrosis	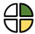	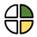	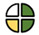	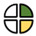	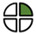
Multifocal necrosis	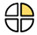	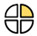	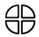	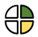	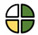
Liver					
Hepatocellular hypertrophy	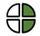	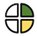	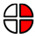	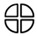	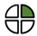
Necrosis of hepatocytes	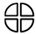	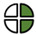	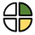	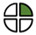	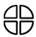
Neutrophilic infiltration	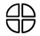	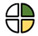	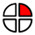	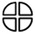	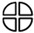
Congestion	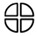	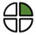	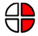	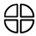	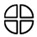
Spleen					
Decreased cellularity in the red pulp	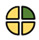	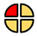	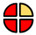	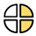	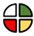
Decreased cellularity in the white pulp, increased apoptosis of lymphocytes in mantel zone	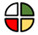	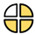	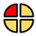	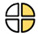	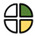
Increased cellularity of FRCsand macrophages in red pulp/stroma	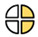	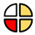	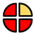	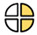	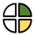
Connective tissue hyperplasia, deposition of collagen fibers in red pulp/stroma	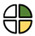	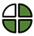	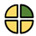	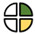	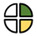
Increase in megakaryocytes in red pulp	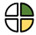	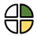	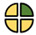	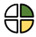	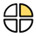

A quarter-circle represents one mouse with mild (green), moderate (yellow), or severe (red) histopathological alterations. The white quarter-circle represents a mouse with no significant alterations. FRCs, fibroblastic reticular cells; PBS, phosphate buffered saline.

**Table 2:** The analysis of biochemical parameters of mice treated subcutaneously with enoxaparin (ENX) (5 mg/kg), and an intravenous bolus of vehicle, HBC (6.25 and 12.5 mg/kg), or protamine sulfate (PS) (5 and 10 mg/kg). The blood for biochemical parameters was drawn from the heart 10 h after the administration of antidotes.

Parameters (Unit)	ENX + PBS	ENX + HBC 6.25 mg/kg	ENX + HBC 12.5 mg/kg	ENX + PS 5 mg/kg	ENX + PS 10 mg/kg
ALT (U/I)	25 (20–28)	40 (25–179) *	192 (40–4715) **	25 (19–46)	35 (25–83) *
AST (U/I)	142 (131–151)	207 (127–353)	1311 (220–2516) *	126 (114–920)	235 (131–375)
Urea (mg/dL)	58 (37–133)	89 (55–91)	102 (59–120)	52 (36–109)	46 (36–120)
Creatinine (mg/dL)	<0.46	<0.46	<0.46	<0.46	<0.46
Albumin (g/dL)	3.4 (3.3–3.8)	4.0 (3.5–4.6) *	3.9 (3.8–4.1) *	3.7 (2.9–3.8)	3.7 (3.3–4.0)
Globulin (g/dL)	1.0 (0.8–1.3)	1.2 (0.9–1.4)	1.4 (1.3–1.7) *	1.1 (1–1.4)	1.1 (0.7–1.3)
Sodium (mmol/L)	151 (148–157)	152 (150–157)	155 (153–160)	153 (147–156)	152 (150–159)
Potassium (mmol/L)	4.5 (4.2–5.5)	4.2 (3.8–5.7)	3.9 (3.8–5.1) *	5.1 (4.6–5.7)	4.8 (4.2–6.4)
Total bilirubin (mg/dL)	<0.15	0.15 (<0.15–0.21)	0.15 (<0.15–0.26)	0.15 (<0.15–0.18)	<0.15
Cholesterol (mg/dL)	59 (54–70)	68 (63–86)	81 (68–125) *	65 (38–87)	63 (53–77)
LDH (U/I)	386 (280–470)	510 (395–579)	1415 (757–5183) *	404 (217–1150)	436 (414–652)
Triglycerides (mg/dL)	45 (35–53)	55 (49–59)	57 (42–68)	49 (15–73)	56 (40–67)
CK (U/I)	157 (128–323)	190 (97–533)	503 (159–6786) *	177 (120–2677)	225 (151–342)

ALT, alanine transaminase; AST, aspartate transaminase; LDH, lactate dehydrogenase; CK, creatine kinase. Results are shown as a median with lower and upper limits. * *p* < 0.05, ** *p* < 0.01 vs. ENX + PBS (phosphate buffered saline), Mann–Whitney test.

## Data Availability

The data presented in this study are available on request from the corresponding author.
